# Prediction of attributable mortality in pediatric patients with cancer admitted to the intensive care unit for suspected infection: A comprehensive evaluation of risk scores

**DOI:** 10.1002/cam4.6709

**Published:** 2023-11-27

**Authors:** Zachary Rubnitz, Yilun Sun, Asya Agulnik, Pamela Merritt, Kim Allison, Jose Ferrolino, Ronald Dallas, Li Tang, Joshua Wolf

**Affiliations:** ^1^ Department of Internal Medicine University of Utah School of Medicine Salt Lake City Utah USA; ^2^ Department of Biostatistics St. Jude Children's Research Hospital Memphis Tennessee USA; ^3^ Department of Global Pediatric Medicine St. Jude Children's Research Hospital Memphis Tennessee USA; ^4^ Department of Infectious Diseases St. Jude Children's Research Hospital Memphis Tennessee USA; ^5^ Department of Pediatrics University of Tennessee Health Science Center Memphis Tennessee USA

**Keywords:** children, infection, neoplasm, prediction, sepsis

## Abstract

**Objective:**

To evaluate the performance of existing sepsis scores for prediction of adverse outcomes in children with cancer admitted to the ICU with suspected sepsis.

**Design:**

Retrospective chart review using data available at 1, 6, 12, and 24 h after ICU admission to calculate the Pediatric Risk of Mortality 3 (PRISM‐3), Pediatric Sequential Organ Failure Assessment (pSOFA), Paediatric Logistic Organ Dysfunction 2 (PELOD‐2), and Quick Pediatric Sequential Organ Failure Assessment (qSOFA) scores.

Area under the receiver operator characteristic curve (AUROC) was used to evaluate performance for prediction of attributable mortality. Sensitivity analyses included recalculation of scores using worst preceding values for each variable, excluding hematologic parameters, and prediction of alternative outcomes.

**Setting:**

St. Jude Children's Research Hospital, a pediatric comprehensive cancer center in the USA.

**Patients:**

Pediatric patients (<25 years of age) receiving conventional therapy for cancer admitted to the ICU with suspected sepsis between 2013 and 2019.

**Results:**

Of 207 included episodes of suspected sepsis, attributable mortality was 16 (7.7%) and all evaluated sepsis scores performed poorly (maximal AUROC of 0.73 for qSOFA at 1 and 24 h). Sensitivity analyses did not identify an alternative approach that significantly improved prediction.

**Conclusions:**

Currently available sepsis scores perform poorly for prediction of attributable mortality in children with cancer who present to ICU with suspected sepsis. More research is needed to identify reliable predictors of adverse outcomes in this population.

## 1 BACKGROUND

Infection and associated sepsis are important causes of morbidity and death in children with cancer.^1^, ^2^, ^3^, ^4^, ^5^, ^6^, ^7^ The high risk of sepsis is related to infections associated with immunocompromise, mucosal barrier damage from chemotherapy, central venous catheters and other interventions, and with immune dysregulation from chemotherapy or cancer itself.^8^ Consequently, around 10%–15% of all pediatric patients with sepsis have an underlying neoplastic condition.^9^, ^10^ Of pediatric patients who develop sepsis, the risk of mortality is also significantly higher in those with cancer (aOR ~2).^9^, ^11^ Lastly, even nonfatal sepsis may have long‐term complications in this population, including excess neurocognitive dysfunction, and bone and joint damage.^12^, ^13^

Several pediatric sepsis risk scores that predict adverse outcomes within the first 24 h after ICU admission have been developed and validated in the general pediatric population.^11^, ^14^, ^15^, ^16^, ^17^, ^18^, ^19^, ^20^ These scores can accurately classify the risk of mortality, length of stay (LOS) and other important complications, and are useful for stratifying participant risk in research studies, selecting patients for more intensive interventions, and counseling caregivers.^11^ However, although these risk prediction scores (including PRISMIII, PELOD‐2, pSOFA, and qSOFA) are highly accurate in the general pediatric population, their performance in the oncology domain is unknown as children with cancer comprised only about 0.5% of participants in relevant studies.^11^

In this study, we aimed to evaluate the performance of four previously‐validated pediatric sepsis risk prediction scores in pediatric patients with cancer admitted to the ICU with suspected sepsis.

## 2 METHODS

This was an institutional review board‐approved retrospective study conducted at St. Jude Children's Research Hospital, a quaternary pediatric cancer center in Memphis, Tennessee. Inclusion criteria included: Age ≤ 24 years, undergoing treatment for cancer at St. Jude, admitted to admitted to the ICU with suspected sepsis between 2013 and 2019. Participants were excluded if their most recent therapy for malignancy was hematopoietic cell therapy at the time of admission (HCT; transplantation or CAR‐T therapy) but were included if they were receiving therapy for relapsed/recurrent malignancy after HCT. Potentially eligible participants were identified from an institutional database, and participant data for this convenience cohort were abstracted from the electronic medical record into a study‐specific database.^21^, ^22^ Suspected sepsis was defined as: collection of a blood culture plus initiation or modification of antibiotic therapy on the same day as admission to ICU. Episodes of suspected sepsis with onset in the ICU or without documented recovery of sepsis‐associated organ function from a prior episode were excluded.

The following scores were calculated at 1, 6, 12, and 24 h after ICU admission: Pediatric Risk of Mortality 3 (PRISM‐3), Pediatric Sequential Organ Failure Assessment (pSOFA), Paediatric Logistic Organ Dysfunction 2 (PELOD‐2), and Quick Pediatric Sequential Organ Failure Assessment (qSOFA).^14^, ^15^, ^16^, ^17^, ^18^, ^19^, ^20^ The most recent available values for each variable were to calculate the scores at each timepoint. Because there is variability in published approaches to calculating these scores, three different schemas were used to calculate each of pSOFA^16^, ^19^, ^20^ and qSOFA,^14^, ^17^, ^23^ and the schema with the highest average AUROC was used for subsequent analyses. Because PaO_2_ was often unavailable, and availability bias is likely to confound results, SaO_2_ was used as a surrogate.^16^ Other missing data were assumed to be normal. Sensitivity analyses included: calculation of each score with worst, rather than most recent, preceding data, and exclusion of hematologic parameters from each score, as these can be related to chemotherapy for malignancy.

The primary outcome measure was attributable mortality, which was defined *a priori* as death within 60 days, or prior to discharge from ICU, without complete recovery of sepsis‐associated organ dysfunction.^24^ Secondary outcome measures included all‐cause mortality within 60 days or prior to discharge from ICU, definitely attributable mortality (death within 60 days, or prior to discharge from ICU, without any recovery of sepsis‐associate organ dysfunction, in contrast to attributable mortality which allowed incomplete recovery), and length of ICU stay.

The performance of each score for the prediction of mortality was evaluated by estimating the area under the receiver operating characteristic curve (AUROC), and comparing between scores and timepoints. The same approach was used to evaluate the performance of each score for prediction of ICU LOS >7 days. Delong tests were conducted to compare AUROC and *p*‐values were adjusted using the Benjamini and Hochberg method to control the false discovery rate. Data analyses were performed using R software, version 4.3.1.

## 3 RESULTS

There were 207 included episodes of ICU admission for suspected sepsis in 166 participants during the study period (Table 1). Median age at presentation to ICU was 13.0 years (IQR 6.3–16.8). The malignancy was classified as relapsed or refractory in 96 (46.4%) episodes, and 127 (61.4%) episodes occurred in participants with hematological malignancies, 54 (26.1%) with solid tumors, and 26 (12.6%) with brain tumors. Median ICU LOS was 3 days (IQR 2–5). Attributable mortality was observed in 16 (7.7%) episodes, any mortality in 22 (10.6%), and definitely attributable mortality in 11 (5.3%). Attributable mortality was ascribed to multiple organ failure in eight episodes, respiratory failure in three episodes, encephalopathy in three episodes, and septic shock or secondary infection in one episode each. As shown in Figure S1, attributable mortality events were distributed throughout the follow‐up period, with 5 (31%) occurring within the first week after ICU admission.

**TABLE 1:** Characteristics of episodes of suspected sepsis.

Characteristic	Attributable mortality (*N* = 16)		No attributable mortality (*N* = 191)	
	*n*	(%)	*n*	(%)
Age in years, median (IQR)	14.1	(12.1–15.6)	13.0	(5.2–16.8)
Sex				
Female	7	(43.8%)	79	(41.4%)
Male	9	(56.3%)	112	(58.6%)
Race (self‐reported)				
White	13	(81.2%)	138	(72.3%)
Black/African‐American	3	(18.8%)	32	(16.8%)
Asian	0		5	(2.6%)
Native American	0		1	(0.5%)
Other/multiple	0		15	(7.9%)
Malignancy type				
Hematologic	12	(75%)	115	(60.2%)
Brain tumor	1	(6.3%)	25	(13.1%)
Solid tumor	3	(18.8%)	51	(26.7%)
Malignancy status				
Relapsed or refractory	11	(68.8%)	85	(44.5%)
All others	5	(31.3%)	106	(55.5%)
HCT status		(0%)		(0%)
Past HCT	1	(6.3%)	10	(5.2%)
No past HCT	15	(93.8%)	181	(94.8%)
ANC at ICU presentation				
<100 cells/μL	9	(56.3%)	99	(51.8%)
≥100–<500 cells/μL	4	(25%)	26	(13.6%)
≥500 cells/μL	3	(18.8%)	66	(34.6%)
WBC at presentation to ICU				
<1000 cells/μL	10	(62.5%)	115	(60.2%)
≥1000 cells/μL	6	(37.5%)	76	(39.8%)

**FIGURE 1:**
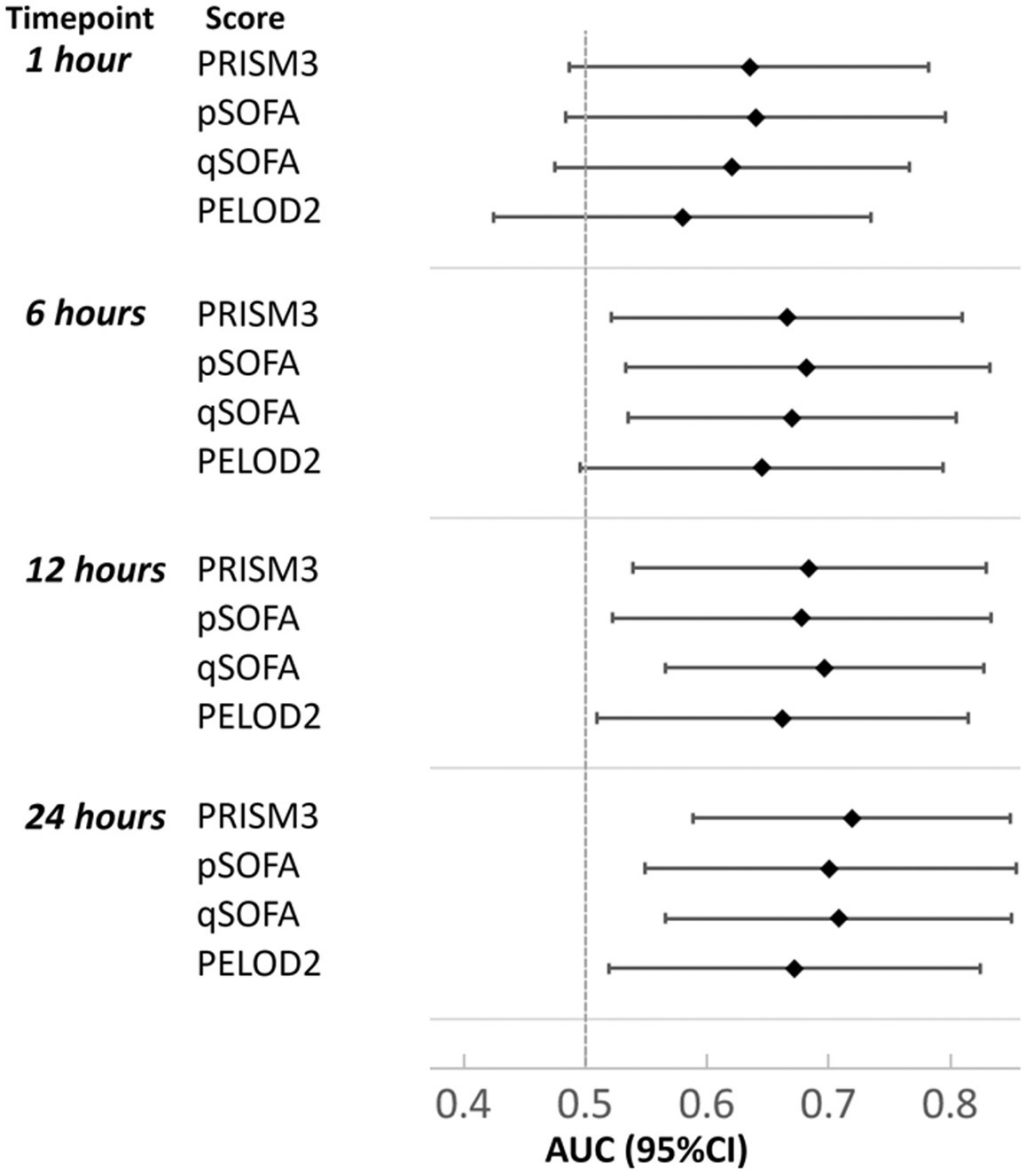
Performance of four pediatric sepsis risk prediction scores for prediction of attributable mortality in children with cancer.

All sepsis scores had poor to moderate discrimination for prediction of attributable mortality, especially early after ICU admission, with a maximal AUROC of 0.73 for qSOFA at 1 and 24 h after ICU admission (Figure 1). The calculation schemas that provided the highest average AUROC for pSOFA^19^ and qSOFA^17^, ^23^ were used for all analyses (Figure S2). Calculation of scores using worst preceding data or excluding hematologic parameters (Table S1; Figure S3) did not significantly improve discrimination. Prediction of any mortality (Figure S4), definitely attributable mortality (Figure S5) or ICU LOS >7 days (Figure S6) were similarly poor, with maximal AUROCs of 0.73, 0.75, and 0.74 respectively. An additional post hoc sensitivity analysis evaluating test performance in participants without relapsed or refractory cancer, comprising 111 participant‐episodes with five attributable deaths, found similar results (Figure S7).

## 4 DISCUSSION

Reliable early predictors of adverse sepsis outcomes are important to allow enrollment in research studies, guide escalation of therapy, and provide prognostic information to patients and caregivers. In this study, several previously‐validated sepsis scores failed to accurately predict attributable mortality in a cohort of children receiving therapy for cancer admitted to ICU with suspected sepsis. Other important findings of the study include a moderate rate of attributable mortality, and a relatively high proportion of delayed attributable mortality.

There are some limitations to this study. The retrospective nature of the data collection means that there may be some misclassification of exposures and outcomes or the presence of unmeasured confounders. Data were missing in more than 10% of episodes for blood gas measurements, coagulation studies, and urine output measurement; this mostly affects calculation of the PRISM‐3 score, so could underestimate performance in this population. Attribution of mortality is challenging in this population because they are at high risk of recurrent infection, cancer‐related death, and other life‐threatening toxicities from cancer therapy. In fact, although many of the participants with attributable mortality experienced secondary infection, it is still reasonable to attribute death to the original episode since subsequent complications occurring prior to recovery from sepsis‐associated organ dysfunction can be related to this persistent organ dysfunction or the required interventions. Sensitivity analyses did not show any important differences using different outcomes. Even if the use of a convenience sample might lead to inaccuracy in the estimating the absolute risk of specific outcomes, it was necessary because a very large number of datapoints was collected for each episode, and should not affect the measured performance of the scores, since this is independent of prevalence. Lastly, because no adjustment was made for multiple comparisons, significant findings may be considered hypothesis‐generating, and require confirmation in an independent cohort. Patients receiving care following HCT were excluded from this study, but are at high risk of sepsis and sepsis‐related mortality; further research is needed to evaluate performance of sepsis scores in that population.

One possible explanation for the poor performance of the scores is the high rate of delayed attributable mortality in this population. In studies of sepsis mortality in the general pediatric population, most attributable deaths occurred within 7 days of presentation and were due to refractory shock or multiple organ dysfunction syndrome.^24^, ^25^ A recent study of pediatric patients with leukemia and sepsis requiring ICU care in Guandong, China found that pSOFA, pediatric early warning score, and pediatric critical illness score had reasonable predictive performance for in‐PICU mortality (AUROC 79%–83%). In contrast to the present study, which had a low attributable mortality of 7.7% with 31% occurring within 7 days, that study had a higher mortality rate (36%), and deaths occurred early in the illness with a median PICU stay of 3 days in non‐survivors. In our study, mortality in many cases was likely caused by secondary infections or other complications related to ICU care without recovery from the original insult. Therefore, these scores may perform better in in children with cancer in low‐ or middle‐income countries, where the primary mode of death is acute non‐survivable organ failure.^7^, ^24^, ^25^ Inclusion in this study cohort did not require a clinical diagnosis of severe sepsis or septic shock, just ICU admission with suspected infection, so the scores might perform better in patients with clearly established sepsis. This suggests that speed of recovery from organ damage might be a better predictor of mortality than initial severity of illness in this population. However, since this information is not available at the time of presentation, a more guarded approach must be taken early on.

Another surprising finding in this study is that attributable mortality was rarely related to withdrawal of care due to refractory malignancy.^24^, ^25^ This is important because it is a reminder that sepsis‐related deaths in pediatric patients with cancer are an important contributor to overall mortality, and are not simply a surrogate for incurable cancer. This is consistent with previous studies that show infection is an important cause of death in front‐line therapy for ALL and AML and reinforces the importance of preventing serious infections in these patients.^2^, ^3^, ^4^, ^26^, ^27^, ^28^

Some potential predictors were not evaluated in this study. A recent study showed that a persistent inflammation, immunosuppression and catabolism syndrome (pPICS) phenotype was an important predictor of mortality and seemed to be enriched in patients with cancer.^29^ Similarly, multiorgan dysfunction phenotype as a binary or multilevel variable might be a more accurate predictor. Lastly, specific biomarkers such as lactate, CRP, ferritin, procalcitonin, or IL‐8, might be more predictive than these scores, but were not reliably available at specific timepoints in this retrospective study.^30^, ^31^, ^32^, ^33^

## 5 CONCLUSIONS

Attributable mortality in children with cancer presenting to ICU with suspected sepsis at a pediatric comprehensive cancer center occurred over a prolonged period after presentation. All available pediatric sepsis scores showed poor to moderate discrimination, even at 24 h after admission. More research is needed to identify factors that best predict adverse outcomes in this uniquely vulnerable population.

## AUTHOR CONTRIBUTIONS

**Zachary Rubnitz:** Data curation (equal); investigation (equal); methodology (equal); writing – original draft (equal); writing – review and editing (equal). **Yilun Sun:** Data curation (equal); formal analysis (equal); methodology (equal); writing – original draft (equal); writing – review and editing (equal). **Asya Agulnik:** Conceptualization (equal); methodology (equal); writing – review and editing (equal). **Pamela Merritt:** Data curation (equal); validation (equal); writing – review and editing (equal). **Kim J Allison:** Conceptualization (supporting); data curation (supporting); methodology (supporting); supervision (equal); writing – review and editing (equal). **Jose Ferrolino:** Data curation (equal); project administration (equal); writing – review and editing (equal). **Ronald Dallas:** Conceptualization (equal); data curation (equal); supervision (equal); writing – review and editing (equal). **Li Tang:** Conceptualization (equal); data curation (equal); formal analysis (lead); methodology (equal); supervision (equal); validation (equal); visualization (equal); writing – review and editing (equal). **Joshua Wolf:** Conceptualization (lead); data curation (lead); formal analysis (equal); investigation (lead); methodology (lead); project administration (lead); supervision (equal); visualization (supporting); writing – original draft (lead); writing – review and editing (lead).

## FUNDING INFORMATION

No funding was obtained for this manuscript.

## CONFLICT OF INTEREST STATEMENT

All authors have no conflicts of interest with regard to this study.

## ETHICS STATEMENT

This study was approved by the St. Jude Children's Research Hospital Institutional Review Board with waiver of consent.

## Supporting information


**Data S1:** Supporting information

## Data Availability

Deidentified data will be made available on reasonable request.
