# Supplementary material for: Prediction of attributable mortality in pediatric patients with cancer admitted to the intensive care unit for suspected infection: A comprehensive evaluation of risk scores
Source: Cancer Med. 2023 Nov 27;12(23):21287–92. doi: 10.1002/cam4.6709 (PMC10726759; doi:10.1002/cam4.6709)
Supplement: Supplementary file 1 — Data S1: Supporting information [file CAM4-12-21287-s001.docx]

**Supplemental Data**

**Table S1.** Performance of pediatric sepsis risk prediction scores modified to use worst preceding values for each variable or exclude hematologic parameters for prediction of attributable mortality in children with cancer

|  | **1 hour** | | | **6 hours** | | | **12 hours** | | | **24 hours** | | |
| --- | --- | --- | --- | --- | --- | --- | --- | --- | --- | --- | --- | --- |
| **Score characteristics** | **Latest** | **Worst** | **P1** | **Latest** | **Worst** | **P1** | **Latest** | **Worst** | **P1** | **Latest** | **Worst** | **P1** |
| PELOD-2 | 0.57 | 0.58 | >0.999 | 0.61 | 0.64 | >0.999 | 0.71 | 0.66 | 0.893 | 0.69 | 0.67 | >0.999 |
| PELOD-2 excluding hematologic parameters | 0.59 | 0.57 | >0.999 | 0.65 | 0.66 | >0.999 | 0.74 | 0.69 | >0.999 | 0.69 | 0.7 | >0.999 |
| P2 | >0.999 | >0.999 |  | >0.999 | >0.999 |  | >0.999 | >0.999 |  | >0.999 | >0.999 |  |
| PRISM-3 | 0.64 | 0.63 | >0.999 | 0.61 | 0.67 | >0.999 | 0.65 | 0.68 | >0.999 | 0.71 | 0.72 | >0.999 |
| PRISM-3 excluding hematologic parameters | 0.65 | 0.6 | 0.736 | 0.62 | 0.64 | >0.999 | 0.64 | 0.66 | >0.999 | 0.7 | 0.69 | >0.999 |
| P2 | >0.999 | 0.134 |  | >0.999 | 0.137 |  | >0.999 | 0.134 |  | >0.999 | 0.134 |  |
| pSOFA (Matics, et al) | 0.63 | 0.6 | >0.999 | 0.62 | 0.65 | >0.999 | 0.65 | 0.64 | >0.999 | 0.71 | 0.66 | >0.999 |
| pSOFA (Matics, et al) excluding hematologic parameters | 0.6 | 0.52 | 0.134 | 0.59 | 0.59 | >0.999 | 0.66 | 0.59 | 0.779 | 0.69 | 0.62 | 0.262 |
| P2 | 0.603 | 0.057 |  | 0.606 | 0.134 |  | >0.999 | 0.134 |  | >0.999 | 0.134 |  |
| pSOFA (Schlapbach, et al) | 0.66 | 0.64 | >0.999 | 0.67 | 0.68 | >0.999 | 0.68 | 0.68 | >0.999 | 0.72 | 0.7 | >0.999 |
| pSOFA (Schlapbach, et al) excluding hematologic parameters | 0.64 | 0.57 | 0.471 | 0.66 | 0.63 | >0.999 | 0.69 | 0.63 | 0.949 | 0.7 | 0.66 | >0.999 |
| P2 | >0.999 | 0.057 |  | >0.999 | 0.134 |  | >0.999 | 0.134 |  | >0.999 | 0.141 |  |
| pSOFA (Shime, et al) | 0.63 | 0.61 | >0.999 | 0.63 | 0.65 | >0.999 | 0.64 | 0.64 | >0.999 | 0.73 | 0.67 | 0.603 |
| pSOFA (Shime, et al) excluding hematologic parameters | 0.59 | 0.53 | 0.346 | 0.6 | 0.6 | >0.999 | 0.64 | 0.6 | >0.999 | 0.7 | 0.62 | 0.134 |
| P2 | 0.606 | 0.057 |  | 0.603 | 0.134 |  | >0.999 | 0.134 |  | 0.914 | 0.134 |  |
| qSOFA (IPSCC criteria) | 0.58 | 0.55 | >0.999 | 0.54 | 0.62 | 0.878 | 0.63 | 0.65 | >0.999 | 0.61 | 0.66 | >0.999 |
| qSOFA (PALS criteria) | 0.73 | 0.62 | 0.141 | 0.64 | 0.67 | >0.999 | 0.68 | 0.7 | >0.999 | 0.73 | 0.71 | >0.999 |
| qSOFA (PELOD-2 criteria) | 0.55 | 0.54 | >0.999 | 0.52 | 0.62 | 0.262 | 0.59 | 0.6 | >0.999 | 0.64 | 0.62 | >0.999 |

P1 represents p-values obtained from comparing the latest preceding values with the worst preceding values; P2 represents the p-values obtained from comparing the inclusion and exclusion of hematologic parameters in the prediction of attributable mortality; PELOD-2, Paediatric Logistic Organ Dysfunction 2; PRISM-3, Pediatric Risk of Mortality 3; pSOFA, Pediatric Sequential Organ Failure Assessment; qSOFA, Quick Pediatric Sequential Organ Failure Assessment; IPSCC, International Pediatric Sepsis Consensus Conference; PALS, Pediatric Advanced Life Support

**Table S2.** Parameters included in each sepsis scoring system

| **Included parameters** | **PELOD-2** | **PRISM-3** | **pSOFA** | **qSOFA** |
| --- | --- | --- | --- | --- |
| **Neurological** |  |  |  |  |
| Glasgow coma scale | X | X | X | X |
| Pupillary response | X | X |  |  |
| **Cardiovascular** |  |  |  |  |
| Arterial blood pressure | X | X | X | X |
| Vasopressors |  |  | X |  |
| Lactate | X |  |  |  |
| pH |  | X |  |  |
| **Renal** |  |  |  |  |
| Creatinine | X | X | X |  |
| Potassium |  | X |  |  |
| Blood urea nitrogen |  | X |  |  |
| **Respiratory** |  |  |  |  |
| Respiratory rate |  |  |  | X |
| PaO_2_/FiO_2_ or SaO_2_/FiO_2_ ratio | X |  | X |  |
| PaO_2_ |  | X |  |  |
| PaCO_2_ | X | X |  |  |
| Invasive ventilation | X |  | X |  |
| **Hematologic** |  |  |  |  |
| White blood cell count | X | X |  |  |
| Platelet count | X | X | X |  |
| Prothrombin or partial thromboplastin time |  | X |  |  |
| **Hepatic** |  |  |  |  |
| Bilirubin |  |  | X |  |
| **Others** |  |  |  |  |
| Temperature |  | X |  |  |
| Blood glucose |  | X |  |  |

PELOD-2, Paediatric Logistic Organ Dysfunction 2; PRISM-3, Pediatric Risk of Mortality 3; pSOFA, Pediatric Sequential Organ Failure Assessment; qSOFA, Quick Pediatric Sequential Organ Failure Assessment; PaO_2_, arterial partial pressure of oxygen; FiO_2_, fraction of inspired oxygen; PaCO_2_, arterial partial pressure of carbon dioxide; SaO_2_, oxygen saturation of arterial blood (measured by pulse oximeter).

**Figure S1.** Timing of attributable and non-attributable mortality in children with cancer admitted to ICU with suspected sepsis


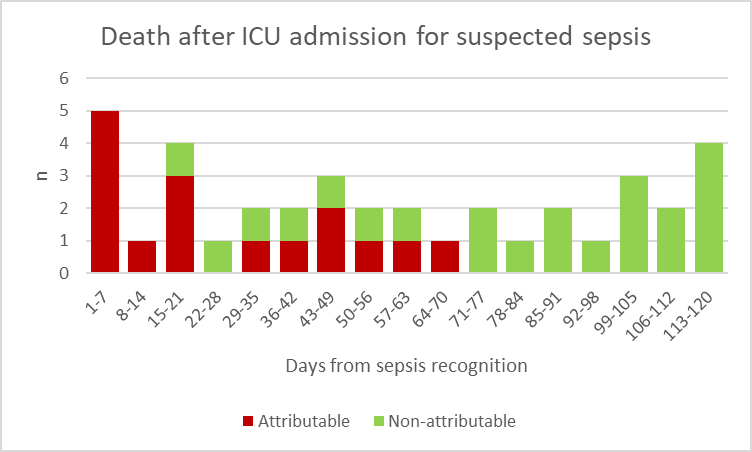


**Figure S2.** Performance of pediatric sepsis risk prediction scores for prediction of attributable mortality in children with cancer

PELOD-2, Paediatric Logistic Organ Dysfunction 2; PRISM-3, Pediatric Risk of Mortality 3; pSOFA, Pediatric Sequential Organ Failure Assessment; qSOFA, Quick Pediatric Sequential Organ Failure Assessment; IPSCC, International Pediatric Sepsis Consensus Conference; PALS, Pediatric Advanced Life Support;
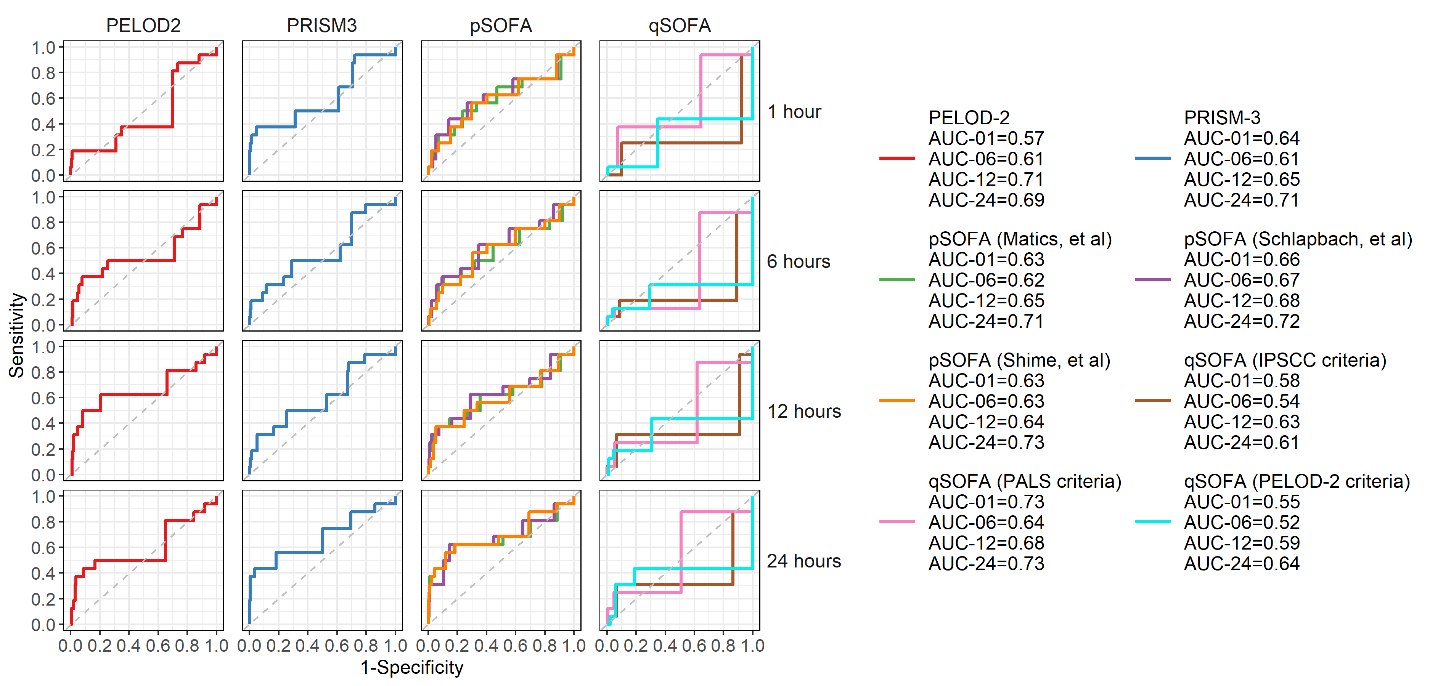
AUC, area under the receiver operating characteristic curve

**Figure S3.** Performance of pediatric sepsis risk prediction scores modified to use worst preceding values for each variable or exclude hematologic parameters for prediction of attributable mortality in children with cancer


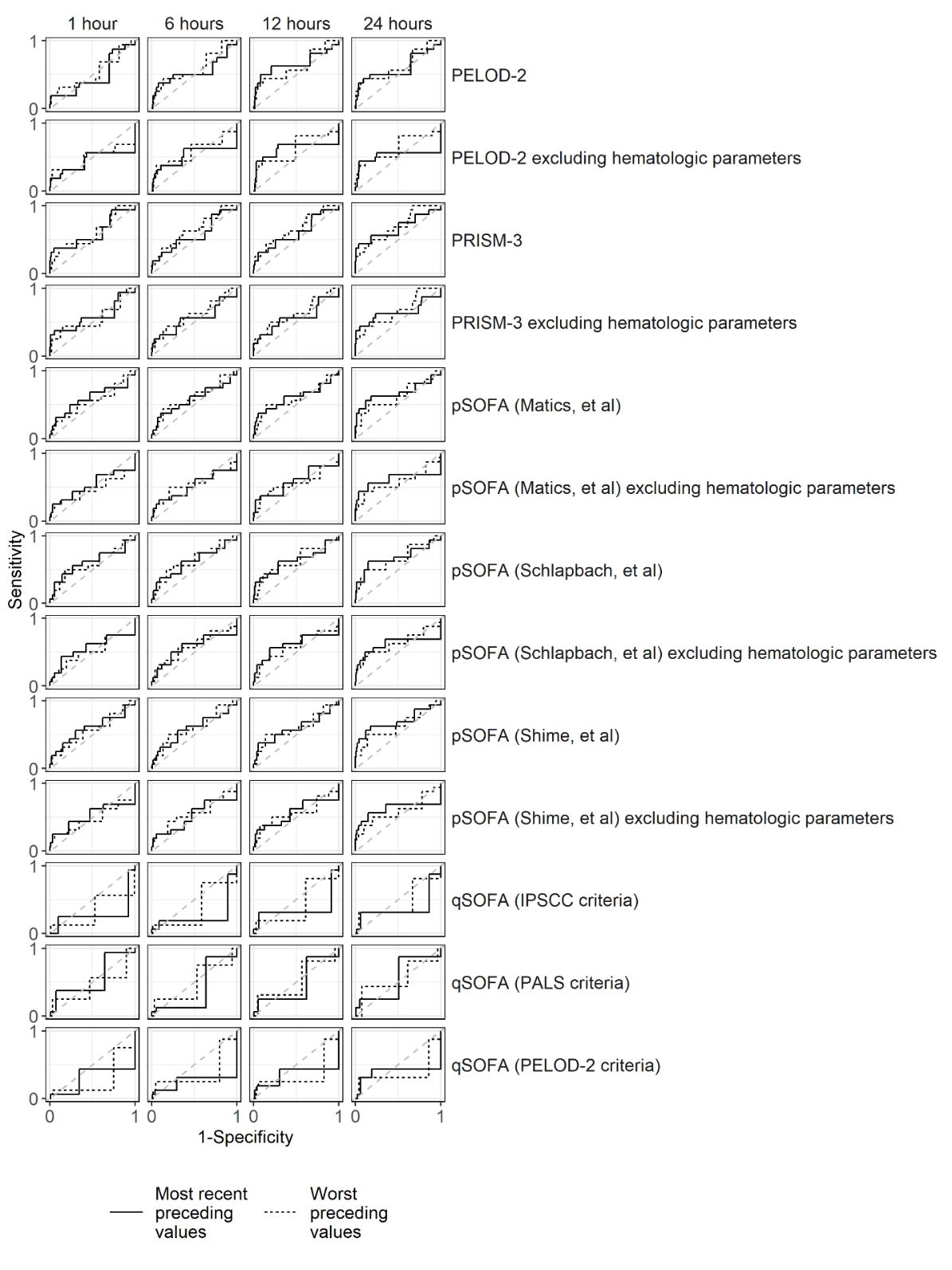


PELOD-2, Paediatric Logistic Organ Dysfunction 2; PRISM-3, Pediatric Risk of Mortality 3; pSOFA, Pediatric Sequential Organ Failure Assessment; qSOFA, Quick Pediatric Sequential Organ Failure Assessment; IPSCC, International Pediatric Sepsis Consensus Conference; PALS, Pediatric Advanced Life Support; AUC, area under the receiver operating characteristic curve**Figure S4.** Performance of pediatric sepsis risk prediction scores for prediction of any mortality in children with cancer

PELOD-2, Paediatric Logistic Organ Dysfunction 2; PRISM-3, Pediatric Risk of Mortality 3; pSOFA, Pediatric Sequential Organ Failure Assessment; qSOFA, Quick Pediatric Sequential Organ Failure Assessment; IPSCC, International Pediatric Sepsis Consensus Conference; PALS, Pediatric Advanced Life Support; AUC, area under the receiver operating characteristic curve
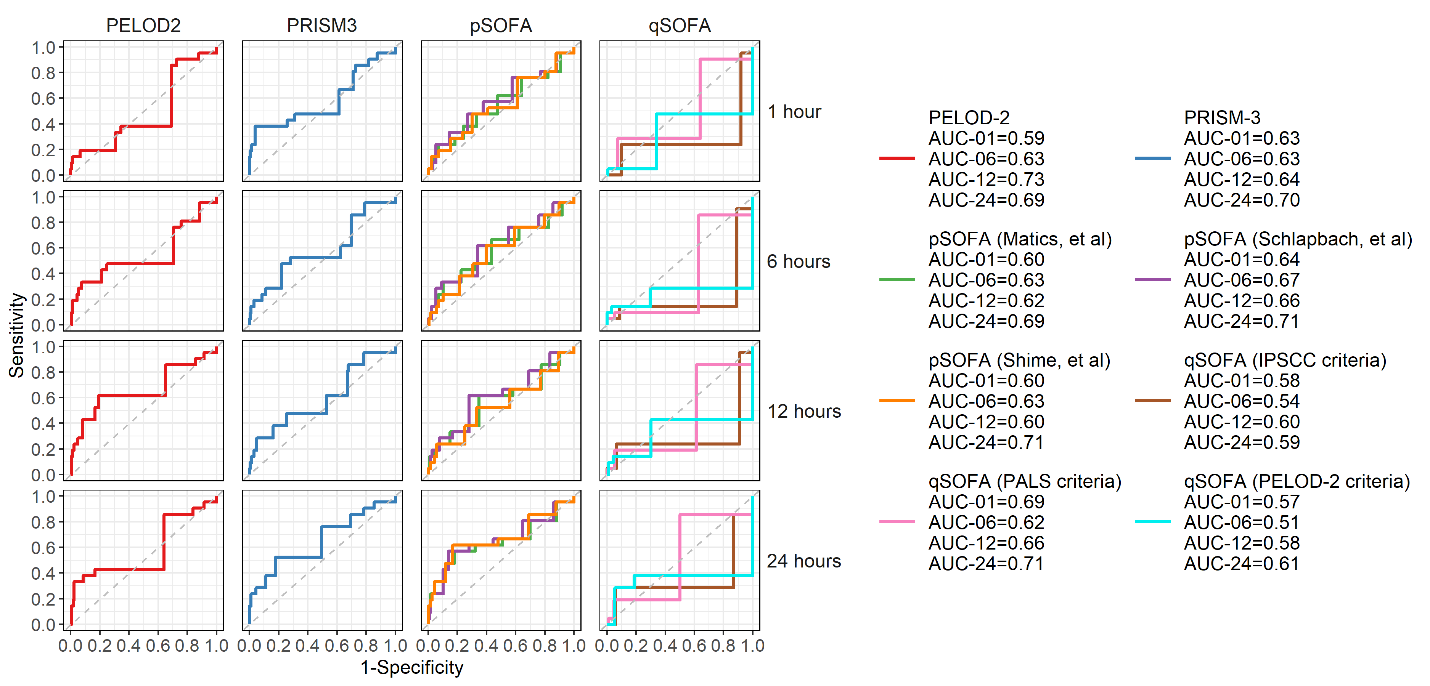


**Figure S5.** Performance of pediatric sepsis risk prediction scores for prediction of definitely attributable mortality in children with cancer

PELOD-2, Paediatric Logistic Organ Dysfunction 2; PRISM-3, Pediatric Risk of Mortality 3; pSOFA, Pediatric Sequential Organ Failure Assessment; qSOFA, Quick Pediatric Sequential Organ Failure Assessment; IPSCC, International Pediatric Sepsis Consensus Conference; PALS, Pediatric Advanced Life Support; AUC, area under the receiver operating characteristic curve
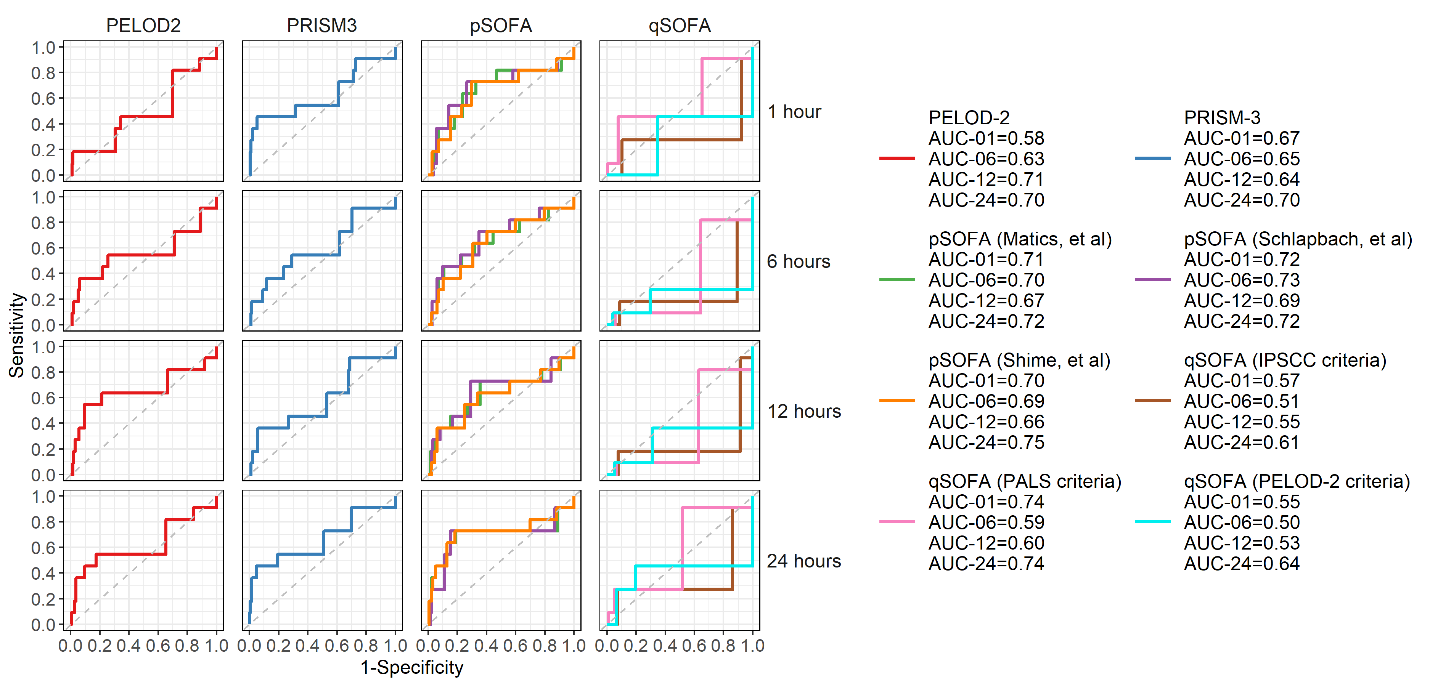


**Figure S6.** Performance of pediatric sepsis risk prediction scores for prediction of prolonged ICU stay (>7 days) in children with cancer


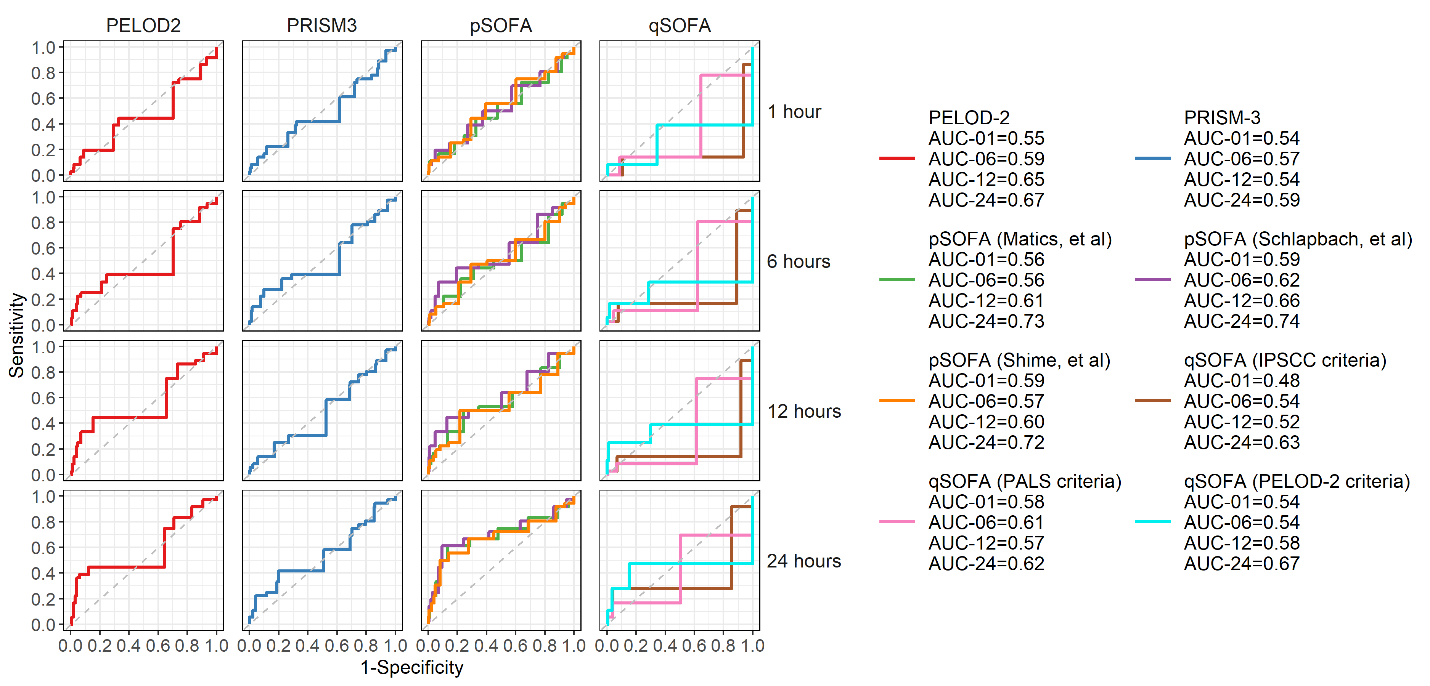


PELOD-2, Paediatric Logistic Organ Dysfunction 2; PRISM-3, Pediatric Risk of Mortality 3; pSOFA, Pediatric Sequential Organ Failure Assessment; qSOFA, Quick Pediatric Sequential Organ Failure Assessment; IPSCC, International Pediatric Sepsis Consensus Conference; PALS, Pediatric Advanced Life Support; AUC, area under the receiver operating characteristic curve

**Figure S7.** Performance of pediatric sepsis risk prediction scores for prediction of attributable mortality in children with cancer excluding participants with relapsed or refractory cancer


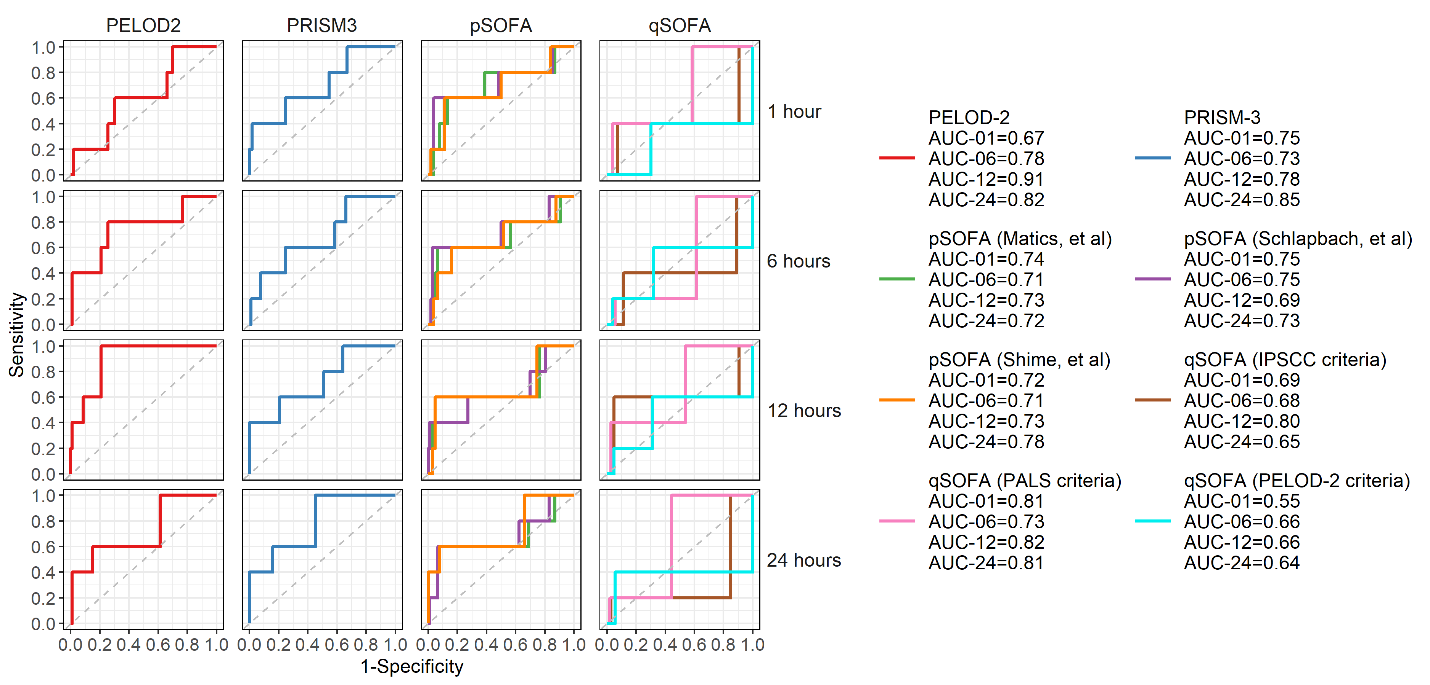


PELOD-2, Paediatric Logistic Organ Dysfunction 2; PRISM-3, Pediatric Risk of Mortality 3; pSOFA, Pediatric Sequential Organ Failure Assessment; qSOFA, Quick Pediatric Sequential Organ Failure Assessment; IPSCC, International Pediatric Sepsis Consensus Conference; PALS, Pediatric Advanced Life Support; AUC, area under the receiver operating characteristic curve
